# Structure and computation-guided yeast surface display for the evolution of TIMP-based matrix metalloproteinase inhibitors

**DOI:** 10.3389/fmolb.2023.1321956

**Published:** 2023-11-23

**Authors:** Alireza Shoari, Ghazaleh Khalili-Tanha, Mathew A. Coban, Evette S. Radisky

**Affiliations:** Department of Cancer Biology, Mayo Clinic, Jacksonville, FL, United States

**Keywords:** matrix metalloproteinase, tissue inhibitor of metalloproteinases, yeast surface display, protein engineering, directed evolution

## Abstract

The study of protein-protein interactions (PPIs) and the engineering of protein-based inhibitors often employ two distinct strategies. One approach leverages the power of combinatorial libraries, displaying large ensembles of mutant proteins, for example, on the yeast cell surface, to select binders. Another approach harnesses computational modeling, sifting through an astronomically large number of protein sequences and attempting to predict the impact of mutations on PPI binding energy. Individually, each approach has inherent limitations, but when combined, they generate superior outcomes across diverse protein engineering endeavors. This synergistic integration of approaches aids in identifying novel binders and inhibitors, fine-tuning specificity and affinity for known binding partners, and detailed mapping of binding epitopes. It can also provide insight into the specificity profiles of varied PPIs. Here, we outline strategies for directing the evolution of tissue inhibitors of metalloproteinases (TIMPs), which act as natural inhibitors of matrix metalloproteinases (MMPs). We highlight examples wherein design of combinatorial TIMP libraries using structural and computational insights and screening these libraries of variants using yeast surface display (YSD), has successfully optimized for MMP binding and selectivity, and conferred insight into the PPIs involved.

## 1 Introduction

The matrix metalloproteinase (MMP) family plays a pivotal role in embryonic development and tissue regeneration. However, the unchecked activity of MMPs is linked to various diseases, including cancer (Gialeli et al., 2011), cardiovascular and pulmonary diseases (Hu et al., 2007; Murphy and Nagase, 2008), rheumatoid arthritis (Grillet et al., 2023), and encephalomyelitis (Clements et al., 1997). Although targeting MMPs presents an attractive therapeutic approach, prior efforts to develop therapeutic MMP inhibitors met with limited success. The primary issue is the similarity of catalytic site structure among MMPs, and consequent poor selectivity of small molecule inhibitors, frequently resulting in musculoskeletal toxicity (Overall and Kleifeld, 2006; Krzeski et al., 2007). This makes the development of highly selective MMP inhibitors a complex challenge.

The engineered TIMPs present a novel alternative to small molecule inhibitors for targeting MMPs in disease (Levin et al., 2017; Radisky et al., 2017). Natural TIMPs are strong protein-based inhibitors of MMPs, and engineered mutations of TIMPs can improve their affinity and selectivity for MMPs (Nagase and Brew, 2002). Furthermore, TIMP inhibitory profiles can be more comprehensively manipulated by using the powerful protein engineering platform, YSD. By leveraging structural knowledge and computational tools, we can design efficient focused libraries, trimming down the vast array of potential mutation combinations (Bonadio and Shifman, 2021). In this review, we highlight recent advances that have employed structure and computation-guided YSD approaches in combination as a cutting-edge method for designing and creating TIMP-based MMP inhibitors.

## 2 Integrating yeast surface display (YSD) with structural and computational design

Combinatorial protein libraries in YSD are presented on the surface of *Saccharomyces cerevisiae* cells; such libraries can contain up to 10^8^ distinct protein variants (Gai and Wittrup, 2007). In comparison to other protein display technologies, the large size of yeast cells enables the use of flow cytometry and fluorescence-activated cell sorting (FACS) to identify antigen-binding cells. YSD typically employs a two-color fluorescence labeling method, where one fluorophore measures antigen binding, while the other measures protein expression (Cherf and Cochran, 2015). This simultaneous approach allows concurrent screening for both affinity and protein stability. Following the screening process, the binding affinity of individual protein variants may also be quantitatively assessed directly on the yeast surface. This efficient workflow allows for rapid prioritization of clones without the need for extensive production and purification of soluble protein for many individual variants (Zahradnik et al., 2021).

A limitation of YSD remains the number of distinct variants that can be generated and screened within a library. While a YSD library may encompass up to 10^8^ variants, this number is dwarfed by the vast sequence space that proteins inhabit. Furthermore, mutations at disparate positions can interact in complex and sometimes unpredictable ways, further complicating the landscape. The limitation in library size thus necessitates efficient library design to make the most of the available screening capacity and ensure that libraries are relevant and enriched in potential solutions (Boder and Wittrup, 1997; Park et al., 2006).

Here, structural insights and computational strategies play a pivotal role. Structural data can highlight key interaction sites, pinpointing regions most amenable to mutations that are likely to affect binding characteristics. Furthermore, computational strategies can simulate and predict the effects of myriad mutations, allowing prioritization of specific high-value sites for diversification, as well as pairs or groups of sites likely to interact. By integrating these structural and computational insights, library design within YSD is elevated to a more strategic level, creating libraries enriched in sequences with the highest potential to achieve desired binding outcomes (Rosenfeld et al., 2016).

After YSD selection, structural and computational methods become essential in interpreting the results. Through next-generation sequencing (NGS), we can gain insights into enriched sequences, revealing preferred mutations and potential interaction domains. Structural analyses of selected variants, combined with molecular modeling, reveal the conformational adjustments and interactions driving enhanced binding (Rosenfeld et al., 2016). Importantly, these insights can be channeled back into refining library designs, paving the way for iterative improvements and increasingly effective protein engineering endeavors.

## 3 Matrix metalloproteinases (MMPs)

MMPs, or matrixins, belong to a larger family of metalloproteinases called the metzincins, which share characteristic structural features of the catalytic domain including a conserved zinc-binding motif at the catalytic site (Cerda-Costa and Gomis-Ruth, 2014; Radisky and Coban, 2021). MMPs degrade different types of extracellular matrix proteins, as well as other substrates including cytokines and cell-surface receptors (Nagase, 1996). These enzymes play a critical role in various biological processes, such as embryogenesis, morphogenesis, and wound healing. Moreover, they participate in pathological processes, such as tumor metastasis, angiogenesis, and inflammation (Malemud, 2006).

### 3.1 MMP classification

MMPs are commonly classified into several groups based on their substrate specificity toward extracellular matrix (ECM) proteins and similarities in their domain structures. Accordingly, MMPs are categorized into gelatinases, collagenases, stromelysins, matrilysins, membrane-type MMPs (MT-MMPs), and others (Table1) (Sternlicht and Werb, 2001; Overall, 2002; Visse and Nagase, 2003; Nagase et al., 2006). All possess N-terminal signal peptides, prodomains, and catalytic domains; other accessory domains vary among the groups.

**TABLE 1:** Members of the human MMP family, and their domains and extracellular substrates (Sternlicht and Werb, 2001; Overall, 2002; Visse and Nagase, 2003; Nagase et al., 2006).

Group/common name	MMP	Extracellular matrix substrates	Domains
Gelatinase			
Gelatinase A	MMP-2	Collagen type I, II, III, IV, V, VII, X, and XI, gelatin, fibronectin, entactin/nidogen-1, decorin, laminin, fibrinogen, aggrecan, fibrillin, elastin, tenascin, vitronectin	Pro-peptide, catalytic domain, fibronectin domain, hinge region, and hemopexin domain
Gelatinase B	MMP-9	Collagen type I, IV, V, XI, and XIV, vitronectin, elastin, aggrecan, decorin, laminin, versican, myelin basic protein, gelatin, fibrin, fibrinogen	Pro-peptide, catalytic domain, fibronectin domain, hinge region, and hemopexin domain
Collagenases			
Collagenase 1	MMP-1	Collagen type I, II, III, VII, VIII, X, and XI, gelatin, entactin, tenascin, aggrecan, fibronectin, vitronectin, myelin basic protein, tenascin, perlecan, fibrin, fibrinogen, laminin 5	Pro-peptide, catalytic domain, hinge region, and hemopexin domain
Collagenase 2 (Neutrophil collagenase)	MMP-8	Collagen type I, II, III, VII, and X, gelatin, fibronectin, aggrecan, entactin, brevican, tenascin	Pro-peptide, catalytic domain, hinge region, and hemopexin domain
Collagenase 3	MMP-13	Collagen type I, II, III, IV, IX, X and XIV, tenascin C isoform, fibronectin, laminin subunit gamma 2, aggrecan, gelatin, fibrinogen, osteonectin, perlecan, biglycan, brevican	Pro-peptide, catalytic domain, hinge region, and hemopexin domain
Stromelysins			
Stromelysin 1	MMP-3	Collagen type I, II, III, IV, V, X, and IX, fibronectin, gelatin, laminin, aggrecan, vitronectin, entactin, tenascin, decorin, myelin basic protein, perlecan, osteonectin, elastin, versican, fibulin	Pro-peptide, catalytic domain, hinge region, and hemopexin domain
Stromelysin 2	MMP-10	Collagen type I, III, IV, and V, proteoglycan link protein 1, gelatin, fibronectin, laminin, elastin, aggrecan, brevican, hyaluronan, fibrinogen	Pro-peptide, catalytic domain, hinge region, and hemopexin domain
Stromelysin 3	MMP-11	Collagen type IV, gelatin, laminin, aggrecan, fibronectin	Pro-peptide, catalytic domain, hinge region, and hemopexin domain
Matrilysins			
Matrilysin 1	MMP-7	Fibulin, versican, fibrin, Fibrinogen, fibronectin, laminin, elastin, osteonectin, gelatin, non-helical segments of native collagen types IV, V, IX, X, XI, vitronectin, entactin, tenascin, aggrecan, myelin basic protein, decorin	Pro-peptide and catalytic domain
Matrilysin 2	MMP-26	Gelatin, native collagen type IV, vitronectin, fibrinogen, fibronectin	Pro-peptide and catalytic domain
Membrane-type MMPs			
A. Transmembrane type			
MT1-MMP	MMP-14	Collagen type I, II and III; gelatin, fibronectin, laminin, vitronectin, galectin-3, fibrillin, tenascin, entactin, aggrecan, lumican, syndecan-1	Pro-peptide, catalytic domain, hinge region, hemopexin domain, type I transmembrane domain, and cytoplasmic domain
MT2-MMP	MMP-15	Laminin, perlecan, fibronectin, entactin, aggrecan, vitronectin, tenascin proteoglycan, myelin basic protein	Pro-peptide, catalytic domain, hinge region, hemopexin domain, type I transmembrane domain, and cytoplasmic domain
MT3-MMP	MMP-16	Gelatin, vitronectin, collagen type III, laminin, fibronectin	Pro-peptide, catalytic domain, hinge region, hemopexin domain, type I transmembrane domain, and cytoplasmic domain
MT5-MMP	MMP-24	Fibronectin, gelatin, dermatan sulphate proteoglycan, chondroitin sulphate proteoglycan	Pro-peptide, catalytic domain, hinge region, hemopexin domain, type I transmembrane domain, and cytoplasmic domain
B. GPI-anchored			
MT4-MMP	MMP-17	Myelin basic protein, fibrinogen, gelatin, fibrin	Pro-peptide, catalytic domain, hinge region, hemopexin domain, and GPI-anchored domain
MT6-MMP	MMP-25	Collagen type IV, fibronectin, gelatin, laminin-1, dermatan sulphate proteoglycan, chondroitin sulphate proteoglycan	Pro-peptide, catalytic domain, hinge region, hemopexin domain, and GPI-anchored domain
Others			
Macrophage metalloelastase	MMP-12	Gelatin, elastin, fibronectin, laminin, vitronectin, biglycan, collagen type I, IV, and V, entactin, osteonectin, aggrecan, fibrin, fibrinogen, decorin	Pro-peptide, catalytic domain, hinge region, and hemopexin domain
RASI-1	MMP-19	Collagen type IV, fibrin, fibrinogen, laminin, nidogen-1, tenascin-C, entactin, aggrecan, fibronectin cartilage oligomeric matrix protein	Pro-peptide, catalytic domain, hinge region, and hemopexin domain
Enamelysin	MMP-20	Ameloblastin, collagen XVIII, aggrecan, laminin, amelogenin	Pro-peptide, catalytic domain, hinge region, and hemopexin domain
Xenopus-MMP	MMP-21	Gelatin	Pro-peptide, catalytic domain, hinge region, and hemopexin domain
Cysteine array (CA-MMP)	MMP-23	Gelatin	Pro-peptide, transmembrane domain, catalytic domain, cysteine array region, IgG-like domain
-	MMP-27	Gelatin	Pro-peptide, catalytic domain, hinge region, and hemopexin domain
Epilysin	MMP-28	-	Pro-peptide, catalytic domain, hinge region, and hemopexin domain

Gelatinases (MMP-2 and MMP-9) can digest gelatin (denatured collagen), some collagens, elastin, fibronectin, and laminin. They possess fibronectin-like repeats as an insertion within in their catalytic domain, which assist in binding to gelatin; they also possess a C-terminal hemopexin domain connected to the catalytic domain by a hinge domain. Gelatinases play key roles in osteogenesis, embryogenesis, angiogenesis, and wound healing. Overexpression of gelatinase was reported in many invasive and metastatic tumors (Nagase et al., 2006; Klein and Bischoff, 2011).

True collagenases (those that efficiently digest intact triple-helical collagen) include MMP-1, MMP-8, and MMP-13, along with MT1-MMP (Amar et al., 2017). C-terminal to the catalytic domain, they possess a hinge domain and hemopexin domain. The collagenases can cleave fibrillar collagen, which is the main component of cartilage and bone (Fischer et al., 2019).

Stromelysins have the same domain organization as collagenases but do not cleave fibrillar collagen, instead digesting a wide variety of collagen fragments and other extracellular matrix components. MMP-3, MMP-10, and MMP-11 belong to this family (Nagase et al., 2006).

Matrilysins (MMP-7 and MMP-26) have the simplest domain structure, comprised of only the signal sequence, prodomain, and catalytic domain. They cleave various components of the extracellular matrix and other substrates. For example, MMP-7 secreted by epithelial cells of the intestine can cleave α-defensin precursors to bactericidal forms, and thus play an important role in innate immunity (Wilson et al., 1999).

Membrane-type metalloproteinases (MT-MMPs) include six MMPs anchored to the plasma membrane. This group can be subdivided into type-I transmembrane proteins including MT1-MMP, MT2-MMP, MT3-MMP, and MT5-MMP, and glycosylphosphatidylinositol (GPI)-anchored proteins including MT4-MMP and MT6-MMP. The type-I transmembrane group have cytoplasmic domains which can play essential roles in cellular signaling (Murphy and Nagase, 2008; Itoh, 2015).

### 3.2 MMP structure

The 3D structures of many MMPs have been revealed by NMR spectroscopy and X-ray crystallography (Maskos, 2005). The prodomain contains ∼88 amino acids and includes a conserved motif (PRCGxPD) containing a cysteine residue which coordinates to the active site Zn ion, maintaining the enzyme in an inactive form (Tallant et al., 2010). Upon proteolytic cleavage between the prodomain and the catalytic domain, the binding between Cys and zinc is disrupted, and the enzyme becomes activated (Ra and Parks, 2007).

The catalytic domain contains the substrate binding cleft and the active site and consists of approximately 170 amino acids (Lovejoy et al., 1994). A conserved sequence, HExxHxxGxxH, consists of three histidine residues interacting with zinc (Bode et al., 1993). There are two zinc atoms; one is catalytic, and the other is structural and additionally, MMPs bind calcium atoms for structural integrity (Massova et al., 1998). The hemopexin-like domain comprises approximately 190 amino acids forming a 4-propeller structure located at or near the C-terminus and is pivotal in interacting with various other proteins (Libson et al., 1995). The hinge region is a flexible linker that differs considerably in length among different MMPs and connects the hemopexin-like domain to the catalytic domain (Nagase et al., 2006). The gelatinases (MMP-2 and MMP-9) additionally possess three fibronectin type II domains inserted within the catalytic domain after the fifth β-sheet (Elkins et al., 2002).

## 4 Tissue inhibitors of metalloproteinases (TIMPs)

Imbalances in production of MMPs and their inhibitors dysregulate ECM turnover and contribute to pathology in various diseases including cancer, cardiovascular diseases, neurological conditions, and arthritis. In healthy tissues, appropriate levels of MMP activity are controlled at multiple levels, including transcriptional regulation, zymogen activation of precursor proteins, localization and binding to specific ECM proteins, and inhibition by endogenous protein inhibitors. TIMPs are the major endogenous inhibitors of MMPs and play a critical role in controlling the balance between the degradation and remodeling of ECM (Murphy and Nagase, 2008).

### 4.1 TIMP classification

Four members of the human TIMP family are encoded by paralogous genes on different chromosomes: TIMP-1 on chromosome Xp, TIMP-2 on chromosome 17p, TIMP-3 on chromosome 22q, and TIMP-4 on chromosome 3p (Murphy, 2011). Orthologs of all four TIMPs are conserved throughout mammals, while homologs are distributed across vertebrate and invertebrate animals (Brew and Nagase, 2010). All four TIMPs inhibit MMPs broadly, but with some differences in affinities toward specific MMPs. For example, MT1-MMP and MMP-19 are only very weakly inhibited by TIMP-1, but more potently inhibited by the other three TIMPs (Batra and Radisky, 2011). All TIMPs are soluble and secreted extracellularly; uniquely, TIMP-3 can also bind tightly to glycosaminoglycans and become incorporated within ECM (Yu et al., 2000; Murphy and Nagase, 2008).

### 4.2 TIMP structure

**FIGURE 1:**
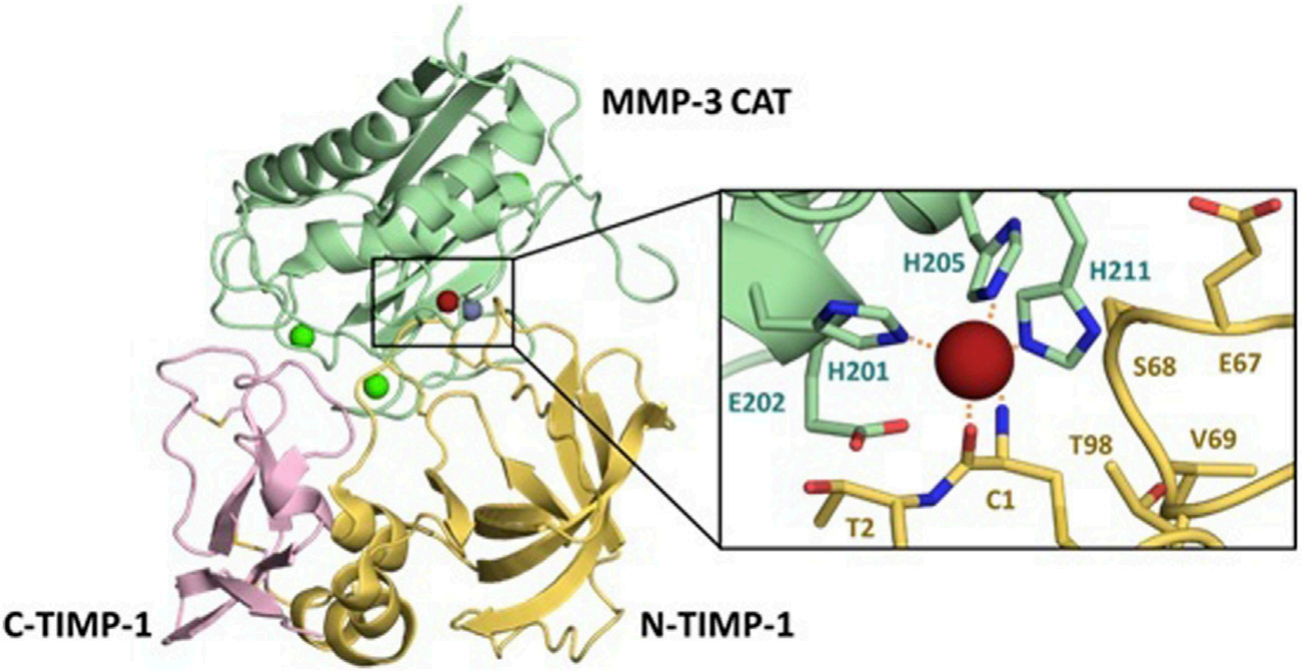
Details of the MMP/TIMP interaction. The MMP-3 catalytic domain (light green) bound to TIMP-1, colored by the N-terminal domain (yellow) and C-terminal domain (pink), illustrates the broad protein-protein interaction interface (PDB ID 1UEA). Structural calciums (bright green), the structural zinc (grey) and catalytic zinc (brick red) are displayed for context. Detail panel shows the conserved zinc-chelating histidines and catalytic Glu202 of the MMP-3 active site, with Cys1 of TIMP-1 chelating the zinc ion.

The TIMP structure is comprised of two domains situated side-by-side (Figure 1) (Batra and Radisky, 2011). The N-terminal domain, also known as the inhibitory domain, consists of approximately 125 amino acids and directly interacts with the zinc ion in the catalytic site, leading to inhibition of catalytic activity. The C-terminal domain has approximately 65 amino acids and for each TIMP may mediate unique interactions with binding proteins or domains distinct from the MMP catalytic domains. For example, TIMP-2 can serve as an adapter protein to assist in the activation of proMMP-2 by MT1-MMP; the TIMP-2 inhibitory domain docks with one catalytic domain of an MT1-MMP dimer, while the TIMP-2 C-terminal domain binds to the hemopexin domain of proMMP-2, bringing the zymogen into proximity with the activating catalytic domain of the second MT1-MMP molecule (Morgunova et al., 2002). TIMPs can also bind to cell-surface receptors and stimulate cell signaling; these activities vary among the four TIMPs. The structure of the N-terminal domain contains a five-stranded β-barrel and three α-helices, while the C-terminal domain has two pairs of β-strands (parallel and antiparallel), which are connected by an α-helix. These domains are about 40% conserved among the four human TIMPs (Batra et al., 2013; Raeeszadeh-Sarmazdeh et al., 2019).

X-ray crystallographic structures of MMP/TIMP complexes reveal that the most crucial protein-protein interactions are made with a conserved ridge on the N-terminal domain of TIMP, which consists of the N-terminal five amino acids Cys1-X-Cys3-X-Pro5, and the C-connector loop which connects β-strands sC to sD. A disulfide bond between Cys1 and Cys70 covalently fastens these two segments, which together fill both the nonprimed and primed subsites of the MMP substrate-binding cleft. The N-terminal amine of Cys1 is positioned at the catalytic site of the MMP, and coordinates to the catalytic zinc ion, displacing the hydrolytic water molecule (Gomis-Ruth et al., 1997). Additional interaction regions which extend the binding ridge are comprised of the TIMP AB-loop of the N-terminal domain and the GH-loop and multiple-turn-loop of the C-terminal domain; these regions have variable involvement in different MMP-TIMP complexes (Brew and Nagase, 2010; Batra and Radisky, 2011; Batra et al., 2013).

## 5 Applications of yeast surface display (YSD) for TIMPs

A primary reason for the failure of MMP inhibitors in clinical trials has been the lack of adequately selective drugs (Clements et al., 1997; Moore et al., 2003; Sparano et al., 2004; Bissett et al., 2005). Therefore, identifying highly selective MMP inhibitors has become a significant goal to improve the effectiveness of these agents and minimize off-target effects. Here, we overview recent work in the field, focusing on novel protein engineering strategies for developing selective TIMP-based therapeutics.

**FIGURE 2:**
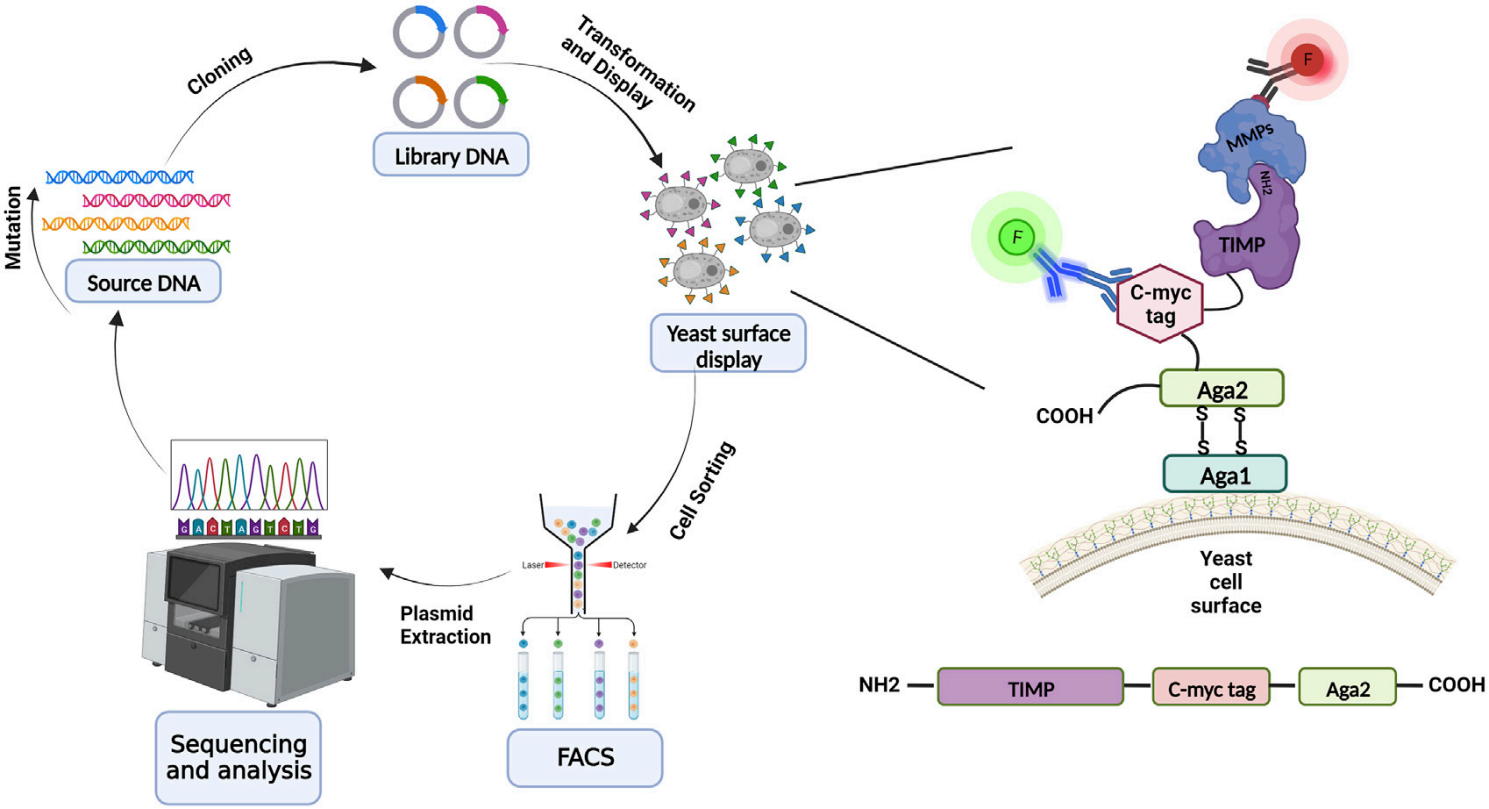
Schematic overview of yeast surface display for library screening and directed evolution. Directed protein evolution cycle utilizing YSD entails producing, expressing, displaying on the yeast surface, and screening libraries, and then sequencing and assessing the isolated variants. TIMP variants are displayed on the yeast surface via fusion at the N-terminus of yeast cell wall protein Aga2; the native N-terminus of the mature TIMP protein must be accessible to enable binding to MMPs during the screening process. TIMP expression is detected through immunolabeling of the c-myc epitope tag, and binding of an MMP is detected using a different fluorescent label (red highlight). (Illustration created with BioRender.com).

Raeeszadeh-Sarmazdeh et al. (2019) screened a large library of full-length TIMP-1 variants displayed on the yeast surface to optimize MMP-3 binding affinity. The library was designed to incorporate diversity across 8 positions in the N-terminal domain and 9 positions in the C-terminal domain, all of which participate in the interaction interface with the MMP catalytic domain (Figure 2). The large number of diversified positions did not allow for comprehensive coverage of sequence-space, but instead allowed broad sampling of variants with multiple mutations (∼4 on average) and enabled us to identify cooperative mutations that work together. Follow-up experiments probed this cooperativity by comparing binding profiles of single and double mutants, while structural studies analyzed the crystal structure of an improved variant to yield insight into the structural basis for binding improvements. Our results showed that the Leu34Gly mutation on the N-terminal domain AB-loop increases the affinity of TIMP-1 to MMP-3 by facilitating formation of a reciprocal clasp between Tyr153 of MMP-3 and Tyr35 of TIMP-1. Moreover, a Gly154Ala mutation on the C-terminal domain changes the conformation of the multiple-turn loop resulting in stronger interaction between two domains and improving MMP-3 binding in comparison with the wild-type TIMP-1 (Raeeszadeh-Sarmazdeh et al., 2019). This study revealed that, despite the common belief that the N-terminal domain of TIMPs is uniquely important for MMP binding and inhibition, the C-terminal domain and the interaction between the two domains can also be crucial in targeting an MMP with the highest affinity.

A subsequent study delved into the challenge of distinguishing between MMP-3 and MMP-10, two MMPs noted for their pronounced structural and functional conservation. Utilizing a counter-selective strategy to screen the library described above, we identified TIMP-1 variants with up to 23-fold improved selectivity for MMP-3 relative to MMP-10 (Raeeszadeh-Sarmazdeh et al., 2022). The two most improved variants each possessed the Leu34Gly mutation, as found in the previous study, along with additional mutations in the N-terminal domain C-connector loop and the C-terminal domain GH- and multiple turn loops. Crystal structures of these TIMP-1 variants in complex with MMP-3 provided insights into how the mutations were accommodated at the binding interface, while structural modeling revealed explanations for losses in affinity toward the counter-target MMP-10. For example, mutations at Met66 in the C-connector loop and Pro131 and Leu133 in the GH loop each disrupt predicted hydrophobic packing interactions with MMP-10, while having less impact on the interaction with MMP-3. Together, these mutations preserved TIMP-1 affinity toward MMP-3 while simultaneously diminishing affinity toward MMP-10 (Raeeszadeh-Sarmazdeh et al., 2022). This study underscores the power of the YSD platform in identifying rare variants that excel in discriminating between closely related targets. Moreover, it emphasizes the indispensable role of structural data and computational modeling in deciphering the underpinnings of enhanced selectivity.

Other efforts to engineer selective TIMPs have employed the more minimal scaffold of the N-terminal domain of TIMP-2 (N-TIMP-2), which retains inhibitory capability toward MMPs. In a seminal study, Arkadash et al. (2017) combined computational approaches for library design with YSD to identify high affinity and selectivity inhibitors of MT1-MMP. First, structural modeling and computational saturation mutagenesis of the TIMP-2 complex with multiple MMPs allowed prediction of binding energy changes that would result from mutations at each site (Sharabi et al., 2014). This systematic analysis identified specific positions at which mutations conferred high probability of improving affinity and specificity toward MT1-MMP. Among these, seven positions were selected for diversification within the focused library: positions 4, 35, 38, 68, 71, 97, and 99 (Arkadash et al., 2017). The top variant identified through YSD screening contained 5 mutations, improved MT1-MMP affinity by approximately 900-fold with a *K*_i_ value of 0.9 pM, and improved selectivity relative to a range of other MMPs by 2–4 orders of magnitude. Structural modeling of key mutations observed in the selected variants lent insight into affinity enhancements toward MT1-MMP. For example, the Ser4Arg mutation ameliorates the interaction of N-TIMP-2 and MT1-MMP by favoring intermolecular hydrogen bonds with Asn231 and Asn229 on MT1-MMP. Mutations of Ile35 to alternative hydrophobic amino acids Met, Pro, or Leu improved packing of MT1-MMP and N-TIMP-2 at the interface. Substitution of Asp at position Asn38 was predominant in selected variants and is predicted to enable formation of a salt bridge with MT1-MMP Lys41, thus stabilizing the interacting loop conformation in the context of the bound complex. The Ser68Asp substitution improves packing and stabilizes the N-TIMP-2 conformation by forming an intramolecular hydrogen bond with Ala70, while the nearby Ile71Val substitution likewise improves packing with MT1-MMP (Arkadash et al., 2017).

Arkadash et al. (2018) subsequently screened the same YSD library of N-TIMP-2 variants using a dual-target screening strategy to obtain selective inhibitors with high affinity for MMP-9 or MT1-MMP. Top variants to emerge from this sorting strategy showed strongly enhanced affinity and selectivity toward the intended targets. The best MT1-MMP-selective variants had ∼200-fold improved affinity toward MT1-MMP, with *K*_i_ values of 24–30 pM. Those selected for targeting MMP-9 showed little change in affinity toward the target, with *K*_i_ values of ∼1 nM, but nevertheless showed significant improvements in specificity. These biochemical results were consistent with the specificity and selectivity of the engineered N-TIMP-2 variants in cell-based assays. In assays with U87MG glioma cells, which depend upon MT1-MMP for activation of MMP-2, the MT1-MMP-selective N-TIMP-2 variant was uniquely able to reduce the activation of MMP-2 by up to 50% (Arkadash et al., 2018). In assays employing MCF-7 breast cancer cells overexpressing MMP-9, the MMP-9-selective N-TIMP-2 was uniquely capable of blocking gelatin degradation and inhibiting cellular migration. Conversely, in MCF-7 cells overexpressing MT1-MMP, the MT1-MMP-selective N-TIMP-2 variant uniquely inhibited cellular migration (Arkadash et al., 2018). These experiments demonstrate that the precision-engineering of TIMPs, guided by the YSD platform, can yield molecules with remarkable target specificity and potency in a cellular context. Moreover, they highlight the potential of tailored protein inhibitors to selectively modulate specific pathways in complex cellular environments, opening avenues for targeted therapeutic applications.

An even more narrowly targeted approach to N-TIMP-2 library design was reported by Shirian et al. (2018), toward a similar goal of developing selective inhibitors for MMP-9 and MT1-MMP. The library incorporated diversity at eight positions computationally predicted to enhance affinity and/or specificity toward the targets (positions 4, 6, 35, 38, 68, 71, 97, and 99). Furthermore, substitutions at each site were restricted to enrich for particular mutations that were shown in previous work to enhance binding specificity toward MT1-MMP as single mutations, resulting in a focused library of 68,480 unique sequences. The dual-target screening strategy employed was similar to that described above, involving simultaneous positive and negative selection with the two targets, each labeled with a different fluorophore. Library screening resulted in identification of distinct specificity signatures for N-TIMP-2 binding to MMP-9 and MT1-MMP. Hallmarks of MMP-9 specificity included Pro at position 4, Asn at position 38, and Ile at position 71, while hallmarks of MT1-MMP specificity included Gln at position 38 and Asn at position 71. The optimized MMP-9-selective N-TIMP-2 variant demonstrated a significant 1000-fold preference towards MMP-9 compared to MT1-MMP. As in the prior studies, computationally modeled structures contributed insights into the mechanisms responsible for conferring the observed shifts in selectivity (Shirian et al., 2018).

Beyond screening combinatorial libraries to extract a few top-performing variants for a particular target, YSD screening can also be combined with computational analyses to comprehensively map binding specificity landscapes of PPIs. For example, Aharon et al. (2020) developed an innovative approach for quantitatively mapping landscapes for interactions between N-TIMP-2 and three MMP targets (MMP-1, MMP-3, and MT1-MMP). The approach employed an N-TIMP-2 YSD library containing all possible single mutations at seven specificity-determining positions of the TIMP/MMP interface (N-TIMP-2 positions 4, 35, 38, 68, 71, 97, and 99). Multi-target library screening into high- and low-affinity populations was combined with advanced NGS analysis to build predictive models capable of comprehensively identifying hot-spots, cold-spots, and specificity-switch mutations that shape affinity and specificity (Aharon et al., 2020). The quantitative binding landscapes describing the interactions between N-TIMP-2 and these three MMPs showed surprising differences, despite the homologous structures of the targets. For example, the binding landscapes for N-TIMP-2/MMP-3 and N-TIMP-2/MMP-1 demonstrated the PPIs to be nearly fully optimized, with the majority of single mutations causing an affinity loss. For N-TIMP-2/MT1-MMP, by contrast, the native PPI was not tuned for optimal affinity, as evidenced by the wide range of binding affinities among mutants, where many mutations had the potential to increase binding affinity (Aharon et al., 2020). As illustrated by this study, integrating post-screening computational analyses with the YSD platform offers a compelling advance, enabling a more holistic understanding of PPIs that may in the future inform the design of TIMPs with yet greater potency and selectivity for specific MMP targets.

Recent efforts to engineer the TIMP scaffold have gone beyond simple residue substitutions, incorporating larger changes into the protein structure. For example, Bonadio et al. (2023) used computational design to incorporate a loop extension into N-TIMP-2, to facilitate new interactions with a nonconserved region of the MMP surface. This design, in which a 7-residue insertion extended the CD-loop, was then used as the basis for generating a focused combinatorial library of N-TIMP-2 variants. In the library, two residues predicted to interact directly with the nonconserved MMP surface patch were fully randomized, along with two loop-neighboring residues, while the five remaining residues of the engineered loop were partially diversified. YSD screening of this library for high affinity to the target MT1-MMP and low affinity to the anti-target MMP-3 was followed by NGS analysis to identify promising variants. The most optimized N-TIMP-2 variant showed *K*_i_ of 29 pM for MT1-MMP and 2.4 μM for MMP-3, a specificity improvement of 7500-fold compared to wild-type N-TIMP-2. By incorporating NGS data into an AlphaFold multiple sequence alignment, high-confidence structural models were generated and then validated via experimental mutagenesis, revealing how the loop extension interacts and discriminates between non-conserved residues on MT1-MMP and MMP-3. This study highlights how computational design and structural modeling can synergize with YSD screening of combinatorial libraries to reshape the basic structure of TIMPs into yet more selective inhibitors of individual MMPs.

Another recent direction in TIMP engineering has been toward the development of bivalent inhibitors capable of simultaneously targeting both an MMP and a second target for enhanced biological effects. In one such study, Yosef et al. (2018) centered their attention on simultaneously targeting synergistic roles of MT1-MMP and integrin αvβ3 in cancer progression. These molecules collaborate to foster invasion, metastasis, and angiogenesis, in part via a sequential activation pathway involving pro-MMP-2 activation by MT1-MMP and subsequent localization at the cell surface by αvβ3 integrin. This work built upon an engineered MT1-MMP-selective N-TIMP-2 variant previously developed by the group (Arkadash et al., 2017), and next used combinatorial library design and YSD screening to evolve a second epitope on N-TIMP2, distal to the MMP interaction site, to selectively bind to αvβ3 integrin. The apex of the N-TIMP-2 B-C loop is known to naturally bind to integrin α3β1 (Seo et al., 2011); here, this epitope was replaced in the combinatorial library with the diversified sequence X-X-X-R-G-D-X-X-X. Screening of the YSD library enabled identification of N-TIMP-2 variants with high affinity for integrin αvβ3 (Yosef et al., 2018). Monovalent and bivalent fusion constructs featuring one or two engineered N-TIMP-2 domains were evaluated in cell-based models of glioblastoma and *in vitro* angiogenesis, to select the top-performing agent, a bi-specific heterodimer targeting both MT1-MMP and αvβ3 integrin. This agent effectively curbed pro-MMP2 activation, invasiveness and capillary tube formation in glioblastoma and endothelial cell models, and reduced tumor growth in nude mice. The assays underscored the benefits of concurrent intervention, while the innovative engineering approach highlights the potential of the TIMP scaffold for bi-specific targeting, not limited to its potent native inhibition of MMP activity.

Another study from Yosef et al. (2021) aimed to produce a multi-specific agent capable of simultaneously intervening in oncogenic processes promoted by MMP-9 and its cell surface receptor CD44. These molecules have complex interactions crucial for cancer progression. MMP-9 not only dimerizes and colocalizes on the cell surface with CD44 through interactions of its hemopexin domain (PEX), but also cleaves CD44 to stimulate cell signaling and further induce MMP-9 expression. Together this interplay boosts cancer cell migration, invasiveness, and facilitates ECM degradation and metastatic potential. Yosef et al. (2021) developed a multi-specific inhibitor that targets both the PEX and catalytic domains of MMP-9 as well as the interaction with CD44 and its downstream oncogenic effects. First, they used the computationally designed N-TIMP-2 library and YSD screening strategy previously described by Arkadash et al. (2017) to identify a high-affinity inhibitor with *K*_i_ of 14 pM for inhibition of MMP-9. Then, they conjugated this inhibitor with PEX using a flexible linker to produce a multi-specific inhibitor that simultaneously targets the MMP9 catalytic domain, the PEX dimerization epitope, as well as CD44. In cell-based assays, the chimeric inhibitor significantly reduced MMP-9 secretion, suppressed MMP-9 gelatinolytic activity, and inhibited the ERK 1/2 phosphorylation that results from interaction of MMP-9 homodimers with CD44 (Yosef et al., 2021). This multi-specific inhibitor represents an advance in TIMP-based antagonists by simultaneously targeting multiple interactions to amplify the potential to disrupt oncogenic functions.

## 6 Conclusion

There has been considerable effort put into generating potent selective inhibitors of disease promoting MMPs, most of which target the catalytic sites of these enzymes (Fields, 2019). Progress has nonetheless been limited, however, due to the challenging constraint of very close structural homology among the 23 human MMP active sites. Three decades after YSD technology emerged, the method has transformed the investigation, engineering, and interpretation of a wide range of protein-protein interactions. Here, we have highlighted recent advancements in which protein engineering of TIMPs through methods combining YSD with structural and computational approaches have enabled creation of TIMP-based MMP inhibitors with enhanced affinity and specificity. The N-TIMP-2 domain has proven to possess a malleable scaffold, tolerant of mutations, for reengineering with altered protease specificity; it is also shown to be a versatile building block for assembly of bivalent and multivalent agents. While the C-terminal domain has been overlooked in most studies, this domain can contribute up to a third of the contact surface area at the TIMP/MMP interface (Batra et al., 2013). The work of Raeeszadeh-Sarmazdeh and colleagues has demonstrated how this interface can be synergistically optimized through structure-based library design and YSD screening to harness the potential of multidomain TIMPs for high-affinity, selective MMP inhibition (Raeeszadeh-Sarmazdeh et al., 2019; Raeeszadeh-Sarmazdeh et al., 2022). We anticipate that further developments in these approaches will continue to expand our understanding of TIMP/MMP specificity landscapes. One specific opportunity includes incorporating more sophisticated analyses of sequence and affinity enrichment data obtained from YSD screens, by harnessing state-of-the-art machine learning methods. Such methods may allow for accurate affinity predictions for complex combinations of mutations not directly observed within a library, thus expanding the size of the sequence space that can be effectively mapped. Another area for development includes the more detailed mapping of interactions of the four native TIMPs with effectors and binding partners beyond the MMP catalytic domains. A better structural understanding of these natural interactions will aid in the targeted ablation of unwanted off-target effects of engineered TIMP-based MMP inhibitors. These advances will ultimately enable creation of a toolbox of selective TIMP probes and inhibitors with therapeutic potential for a wide variety of diseases.
